# Probing Phase Transitions and Interfacial Reorganization in TAPC/CBP/BPhen Organic Light-Emitting Diode Stacks by In Situ Ellipsometry

**DOI:** 10.3390/ma18102261

**Published:** 2025-05-13

**Authors:** Ilze Aulika, Patricija Paulsone, Sven Oras, Jelena Butikova, Margarita Anna Zommere, Elina Laizane, Aivars Vembris

**Affiliations:** 1 Institute of Solid State Physics, University of Latvia, Kengaraga iela 8, LV-1063 Riga, Latvia; patricija.paulsone@cfi.lu.lv (P.P.); jelena.butikova@cfi.lu.lv (J.B.); margarita-anna.zommere@cfi.lu.lv (M.A.Z.); elina.laizane@cfi.lu.lv (E.L.); aivars.vembris@cfi.lu.lv (A.V.); 2 Institute of Technology, University of Tartu, Nooruse 1, 50411 Tartu, Estonia; sven.oras@ut.ee

**Keywords:** phase transitions, interphase layer, spectroscopic ellipsometry, OLED thin films, thermal analysis, crystallization, optical properties, surface morphology

## Abstract

The thermal behavior of a three-layer structure—glass/ITO/TAPC/CBP/BPhen—in an OLED system was investigated using in situ spectroscopic ellipsometry during controlled heating from room temperature to 120 °C over 60 min, simulating the ageing process and analyzing degradation kinetics. Variations in Ψ and Δ spectra were observed across the entire 0.7–5.9 eV spectral range, with five distinct anomalies, particularly in the UV region. An anomaly at approximately 66 °C was attributed to the glass transition temperature *T_g_* of BPhen, while another two at around 82 °C and at around 112 °C corresponded to the first-order phase transition of TAPC and *T_g_* of CBP, respectively. The origins of the remaining anomalies at 91 °C and 112 °C were explored in this study, with a focus on interphase layer formation and morphological changes that emerge during heating. These findings provide insights into the stability of OLEDs under thermal stress.

## 1. Introduction

Organic light-emitting diodes (OLEDs) have transformed display and lighting technologies owing to their exceptional efficiency, mechanical flexibility, and tunable emission characteristics [1]. These devices employ complex multilayer architectures consisting of organic and inorganic materials, where both bulk and interfacial properties critically influence device performance and operational stability. Despite remarkable advancements, the long-term reliability of OLEDs remains a significant challenge, with degradation mechanisms driven by factors such as electrical stress, photo-oxidation, and, importantly, thermal effects.

Thermal stress has increasingly been recognized as a central factor affecting OLED longevity. Swayamprabha et al. [1] demonstrated that elevated temperatures can trigger molecular rearrangements, delamination, and crystallization within the organic layers, leading to charge imbalance and eventual device failure. Moreover, many organic materials used in OLEDs, such as CBP and BPhen, possess relatively low glass transition temperatures *T_g_*, making them especially prone to morphological instability under modest thermal loads [1,2]. Yang and Cao [3] further highlighted how non-uniform heating and localized Joule heating near critical interfaces—particularly in blue OLEDs—can accelerate chemical degradation, exciton–polaron interactions, and interfacial diffusion.

Recent advanced analyses have emphasized the crucial role of interfacial degradation. Sul et al. [4] used high-resolution Orbitrap secondary ion mass spectrometry (OrbiSIMS) to directly identify chemical degradation products localized at the emissive layer (EML) and electron transport layer (ETL) interface with nanometer-scale depth resolution. These findings clearly linked chemical fragmentation to regions with high exciton density, reinforcing the view that interfacial chemistry is a major determinant of device failure. Similarly, Lee et al. [3,4,5] showed that the selection of ETL materials with higher *T_g_* and suitable energy alignment significantly enhances both efficiency and operational lifetime in blue OLEDs.

While numerous studies have investigated OLED degradation [2,3,4,5,6,7,8], most are based on post-aging assessments or electrical stress protocols. Investigations that specifically target thermally induced phase transitions using in situ optical methods remain scarce. CBP and BPhen have been consistently identified as thermally sensitive layers susceptible to exciton-induced and structural degradation. CBP, with a *T_g_* around 62 °C, tends to undergo morphology-driven spectral drift during operation [1,2]. BPhen, often used as an ETL, has also been shown to degrade at elevated temperatures, with reports suggesting that thermal diffusion and phase separation—particularly at the CBP/BPhen interface—can undermine device integrity [6,7].

TAPC, widely used as a hole transport material, is also vulnerable to degradation, especially due to arylamine reactivity under sustained electrical operation [6,8]. However, most of the knowledge about degradation in these materials stems from ex situ methods, including thermal annealing studies and post-mortem characterization [9,10,11], which lack the ability to track dynamic, real-time structural changes under thermal stress.

In this context, in situ spectroscopic ellipsometry (SE) emerges as a valuable, non-destructive technique capable of tracking thermally induced phase transitions and interfacial reorganizations in multilayer OLED structures with sub-nanometer precision. SE has been widely employed to study phase transitions in a range of materials—from polymers and thin films to single crystals—through temperature-dependent variations in the refractive index *n*, extinction coefficient *k*, optical band gap *E_g_*, surface roughness, and ellipsometric angles (Ψ, Δ) [12,13]. Despite its proven capabilities, SE has not yet been systematically applied to multilayer OLED stacks to monitor transitions such as *T_g_* phase reorganization or interfacial mixing in real time.

**Figure 1:**
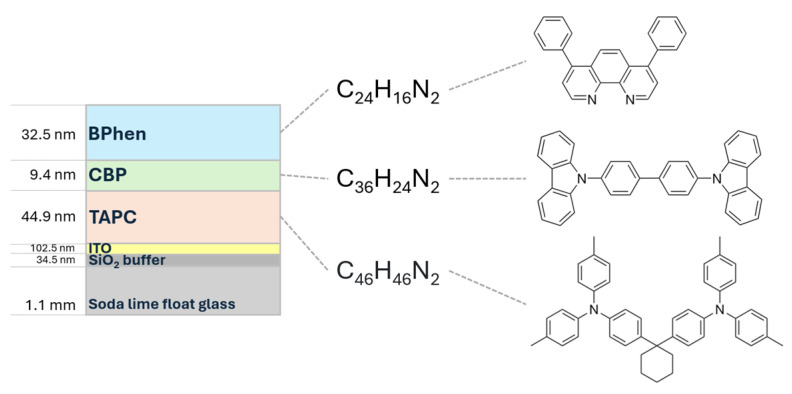
Schematic illustration of a multilayer organic electronic device structure comprising a soda lime float glass substrate (1.1 mm), a SiO_2_ buffer layer (34.5 nm), an indium tin oxide (ITO) anode (102.5 nm), and three organic layers: TAPC (44.9 nm), CBP (9.4 nm), and BPhen (32.5 nm). The molecular structures and chemical formulas of the organic compounds—TAPC (C_46_H_46_N_2_), CBP (C_36_H_24_N_2_), and BPhen (C_24_H_16_N_2_)—are shown on the right, indicating their respective positions within the device architecture.

To address this gap, we employ in situ SE to examine thermally induced transitions in a model OLED multilayer system consisting of soda lime glass/SiO_2_ buffer/ITO/TAPC/CBP/BPhen (Figure 1). Controlled heating from room temperature to 120 °C revealed five distinct anomalies in the Ψ and Δ spectra. The transitions at approximately 66 °C, 82 °C, and 112 °C are attributed to the phase transition or structural reorganizations of BPhen, TAPC, and CBP, respectively. Additional spectral features are interpreted as indicators of interfacial modification and morphological changes—specifically, the formation of interphase regions between CBP and BPhen, and partial crystallization of the organic layers.

This work demonstrates that in situ spectroscopic ellipsometry offers valuable insights into the thermal stability, morphological evolution, and phase behavior of OLED materials under controlled thermal conditions mimicking operational degradation. As such, it offers a complementary and powerful diagnostic tool to support the development of more robust, long-lived OLED devices.

## 2. Materials and Methods

### 2.1. Sample Preparation

Organic compounds were deposited as multilayer thin films on indium tin oxide (ITO)-coated soda lime glass substrate with a sheet resistance of 20 Ω/sq. The glass substrate was covered with a thin SiO_2_ buffer layer for better ITO adhesion. To ensure optimal film adhesion and minimize contamination, the substrate underwent a rigorous multi-step cleaning process. ITO substrate was cleaned by ultrasonic cleaning sequentially in acetone, detergent, deionized water, and isopropanol, with each step lasting 15 min. Afterwards, the substrates were dried using high-purity nitrogen gas. Organic layers were deposited via thermal evaporation in a high-vacuum chamber maintaining 6 × 10^−6^ Torr using a MiniLab LT090A-MX/GP (Concept)-T thermal evaporator. This thermal evaporator is manufactured by Moorfield Nanotechnology Ltd., located in Knutsford, Cheshire, United Kingdom. This model is a glovebox-compatible physical vapor deposition system designed for thin-film deposition of metals, dielectrics, and organic compounds. It supports multiple deposition techniques, including thermal evaporation, low-temperature evaporation, electron-beam evaporation, and magnetron sputtering. The system features a modular design, allowing for customization based on specific research needs.

To achieve a uniform and randomly oriented molecular distribution, the deposition was performed at room temperature. The multilayer structure consisted of three organic compounds, each selected for its distinct functional role within the OLED architecture (Figure 1):

- TAPC (4,4′-Cyclohexylidenebis [N,N-bis (4-methylphenyl)benzenamine])—a hole-transporting material that facilitates charge mobility within the device.
- CBP (4,4′-Bis (N-carbazolyl)-1,1′-biphenyl)—a widely used host material providing an efficient energy transfer environment for emitters.
- BPhen (Bathophenanthroline)—an electron-transport material that improves charge balance within the OLED structure.

The deposition sequence and planned thicknesses were 40 nm for TAPC, 20 nm for CBP, and 40 nm for BPhen. All layers were deposited at a controlled rate of 1 Å/s to ensure uniform film growth. Each of these layers was thermally evaporated in sequence, ensuring precise thickness control and high film uniformity. The obtained thickness values for three organic compounds varied from the planned values: 44.9 nm for TAPC, 9.4 nm for CBP, and 32.5 nm for BPhen. The reduced thicknesses observed for CBP and BPhen may be attributed to their inherently low sticking coefficients, which limit the efficiency of molecule adsorption onto the substrate during thermal evaporation.

A total of three soda lime glass/SiO_2_ buffer/ITO/TAPC/CBP/BPhen samples were fabricated, all exhibiting comparable thickness distributions and optical properties (discussed later). The temperature-dependent behavior was consistent across all samples; therefore, representative results from one sample are presented in this report.

### 2.2. In Situ SE Measurements and Modelling

Ellipsometric measurements were performed using a variable-angle spectroscopic ellipsometer (RC2–XI, J.A. Woollam, Lincoln, NE, USA), covering a broad spectral range from 210 to 1690 nm (0.7 to 5.9 eV). The in situ thermal analysis was conducted at a fixed incident angle of 70°, where the primary ellipsometric parameters, Ψ and Δ, were recorded as a function of the temperature. A controlled heating stage was utilized to systematically increase the sample temperature from room temperature (22 °C) to 120 °C at a uniform heating rate of 2 °C/min, ensuring precise thermal stability and minimizing abrupt structural changes during phase transitions.

The acquired ellipsometric spectra were analyzed using the CompleteEASE^®^ software version 6.73 (J.A. Woollam), where model-based regression techniques were applied to achieve optimal data fitting. The refractive index *n* and extinction coefficient *k* dispersions of the organic thin films were parameterized using the Herzinger–Johs Parameterized Semiconductor (HJPS) model and Gaussian oscillator (GO) functions, as described in our previous work [14]. The (*n*, *k*) dispersion curves for the TAPC, CBP, and BPhen organic compounds are available in [15]. The direct bandgap energy *E_g_* was determined from the oscillator model, as the oscillators include *E_g_* as a fitting parameter in the dispersion analysis.

To ensure reliability, the optical properties of the substrate materials (SiO_2_/Si, buffer SiO_2_/ITO) were independently characterized prior to thin-film deposition [16]. The obtained (*n*, *k*) curves exhibited agreement with the literature values and established material databases in CompleteEASE^®^.

Organic thin films were modelled as optically isotropic materials, with no detectable interfacial layers observed between the films and the substrate before heating. Notably, the most pronounced spectral anomalies in Ψ and Δ were observed in the near-UV and UV spectral regions, correlating with temperature-induced variations in optical properties. The analysis focused on tracking the evolution of the complex refractive index (*n*, *k*), interphase layer formation, surface roughness, and film morphology changes, particularly the development of elongated crystalline islands as a function of temperature. These findings provide critical insights into the thermal-induced phase transitions and morphological transformations occurring in OLED multilayer thin films.

Since the thermal expansion coefficients for BPhen, CBP, and TAPC are approximately 1.0 × 10^−4^ K^−1^, the thickness expansion of these films due to the heating remains minimal. The initial thicknesses at room temperature are 32.54 nm for BPhen, 9.44 nm for CBP, and 44.95 nm for TAPC. As the temperature increases up to 111–120 °C, the expansion in film thickness is estimated to be approximately 0.3–0.5 nm. Given the small magnitude of this change, it can be considered negligible in the context of the overall film properties and optical measurements.

The dynamic thermal modelling was conducted to determine the (*n*, *k*) values at phase transition temperatures and to analyze variations in interphase roughness and patterning as a function of temperature. To establish the optical constants at phase transition points, the dispersion curves were modelled specifically at the corresponding temperatures. Given the complexity of the three-layer OLED structure, which introduced a high number of fitting parameters, an individual fitting approach was necessary for each temperature.

A fully dynamic fitting process incorporating (*n*, *k*) variations for each OLED compound, along with temperature-dependent changes in interphase, roughness, and patterning, was not feasible due to strong parameter correlation detected during the fitting process. Therefore, an independent analysis of (*n*, *k*) at the selected phase transition temperatures was performed for the TAPC and BPhen compounds, ensuring the retrieval of physically meaningful optical constants. Similarly, separate evaluations of interphase, roughness, and patterning effects allowed for a clearer interpretation of anomalies observed at 76 °C, 91 °C, and ~112 °C, which were determined to be unrelated to the phase transitions of BPhen, CBP, and TAPC, but instead attributed to other structural or morphological changes in the OLED system. The (*n*, *k*) dispersion curve at the glass transition temperature *T_g_* of CBP could not be determined due to the low film thickness and the complexity of the optical model. The model required the consideration of multiple factors, including the interphase between CBP and BPhen, surface roughness, and film patterning caused by crystallization, making it challenging to extract reliable optical constants at *T_g_* for the CBP compound.

Due to the irreversible morphological changes in CBP and BPhen upon exceeding their glass transition temperatures—leading to crystallization and partial dewetting (film patterning) of the multilayer structure—the original morphology was not restored upon cooling, and therefore, no cooling data are presented in this study.

The surface roughness and surface quality were analyzed with an atomic force microscope Dimension Edge (AFM Veeco) and optical microscope Nikon Eclipse L150 before and after heating.

## 3. Results and Discussion

**Figure 2:**
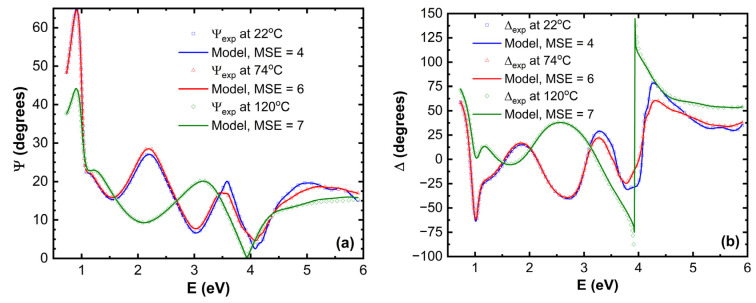
Main ellipsometric angles (**a**) Ψ and (**b**) Δ as a function of photon energy *E* at 22 °C, 74 °C, and 120 °C.

**Figure 3:**
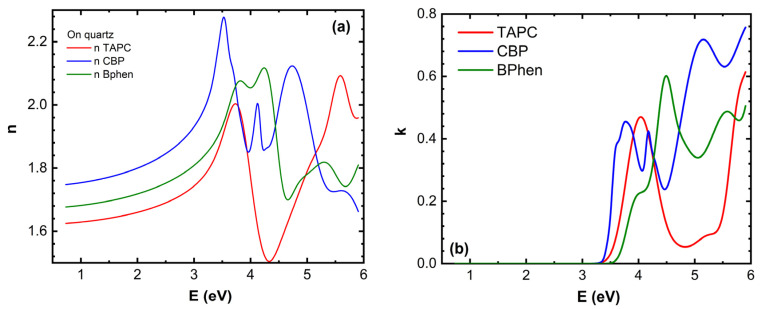
Refractive index *n* (**a**) and extinction coefficient *k* (**b**) as a function of photon energy *E* for the TAPC, CBP, and BPhen compounds at room temperature.

The main ellipsometric angles (Ψ, Δ) as a function of photon energy *E* at three temperatures are presented in Figure 2. A good model fit was obtained for all temperatures. In Figure 2, only room temperature, 74 °C, and 120 °C (Ψ, Δ) spectra are shown. The optical properties of TAPC, CBP, and BPhen are presented in Figure 3.

**Figure 4:**
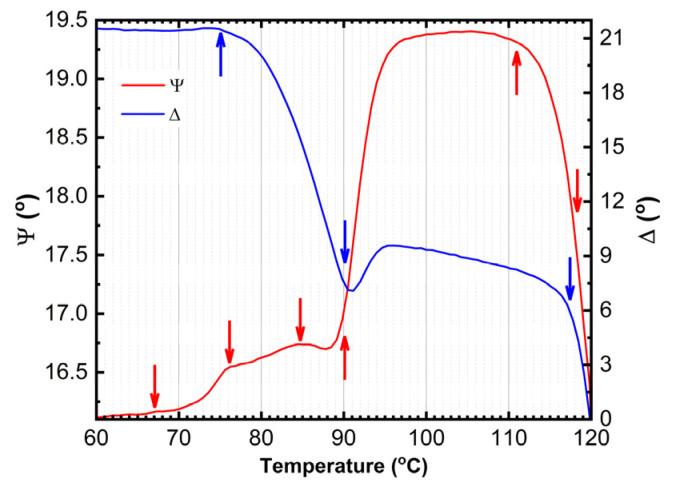
Main ellipsometric angles (Ψ; Δ) as a function of temperature at the photon energy of 3.425 eV.

It is evident that there are significant variations in the (Ψ, Δ) spectra in the UV and near-UV spectra, and a very slight variation in the visible (VIS) spectral regions for the glass/SiO_2_ buffer/ITO/TAPC/CBP/BPhen structure during the heating up to 74 °C. The fact that (Ψ, Δ) varies more in UV and VIS, and the variation is practically absent in the near-infrared (NIR) region, can be explained by the fundamental optical properties of TAPC/CBP/BPhen materials and the manner of light interaction with these compounds at different wavelengths. BPhen, CBP, and TAPC are organic semiconductors with highly conjugated molecular structures, meaning their electronic transitions primarily occur in the UV and VIS range. These materials exhibit strong absorption near their bandgap energies, typically in the UV/near-UV region, as well as significant dispersion in (*n*, *k*), leading to pronounced variations in the ellipsometric parameters (Ψ, Δ). Additionally, excitonic and charge transfer transitions are highly sensitive to the molecular ordering and phase changes, making phase transitions particularly evident in this spectral range. Since phase transitions often involve changes in molecular packing, crystallinity, or electronic structure, they induce distinct modifications in the optical constants (*n*, *k*), which the ellipsometer detects as clear shifts or anomalies in the Ψ and Δ spectra (Figure 4). In contrast, the NIR region corresponds to much lower photon energies, where organic semiconductors like BPhen and TAPC exhibit minimal absorption because electronic transitions in this range are weak or absent. In this spectral region, these materials primarily behave as transparent dielectrics, meaning that (*n*, *k*) spectra show only weak dispersion. Although thermal or phase-induced changes in film morphology, density, or refractive index may still occur, they produce only subtle effects in the NIR region, making phase transitions less evident or even undetectable. Ellipsometry is most sensitive in the spectral regions where a strong contrast in optical properties is observed before and after the transition. The material must exhibit significant variations in (*n*, *k*) due to molecular reorganization or phase changes, and optical absorption must allow for the direct probing of molecular interactions. Since phase transitions in organic materials often influence π-π stacking, crystallization, or molecular orientation, their optical response is most pronounced in the UV and visible ranges, where these transitions occur.

The temperature dependence of the main ellipsometric angles Ψ and Δ is presented in Figure 4, revealing several distinct anomalies at approximately 66 °C, 76 °C, 84 °C, 91 °C, and 112 °C. These anomalies indicate significant thermal-induced changes in the optical properties of the multilayer OLED structure. The anomalies are observed practically in the whole spectral range: more evident in UV, near-UV, and VIS, and less evident or absent in near-IR region.

The observed anomalies at 66.5 °C, 84.5 °C, and 113.0 °C correspond to phase transitions in BPhen, TAPC, and CBP, respectively, as reported in the literature (Table 1). The phase transition of BPhen and CBP are glass transitions, and the transition of TAPC is the reversible first-order phase transition. In contrast, the crystallization temperature *T_cr_* of CBP is known to occur at 205 °C, which is beyond the temperature range of this study, and thus remains unobserved [17].

**Figure 5:**
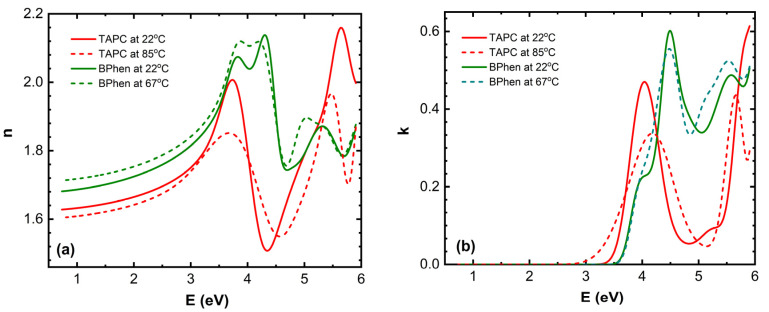
Refractive index *n* (**a**) and extinction coefficient *k* (**b**) as a function of photon energy *E* for the TAPC and BPhen compounds at room temperature and just after phase transition temperature.

The (*n*, *k*) dispersion curves for the TAPC and BPhen compounds at room temperature and just after phase transition temperatures are presented in Figure 5. A slight increase in *n* for BPhen near its *T_g_* (Figure 5a) can be attributed to the subtle densification of the film, which occurs due to structural relaxation or early-stage crystallization. At *T_g_*, the material transitions from an amorphous glassy state to a more fluid-like state, allowing molecular rearrangement and closer packing, which increases the film density. This denser molecular arrangement enhances the polarizability per unit volume, leading to a slight increase in the refractive index. However, if full crystallization occurs, more significant changes in anisotropy and optical dispersion may be observed. The slight decrease in *n* for TAPC during its first-order phase transition (Figure 5a) likely occurs due to a combination of reduced film density, increased molecular disorder, and changes in intermolecular interactions. This structural transformation results in a lower optical polarizability, leading to a decrease in the refractive index.

The peak positions of the extinction coefficient *k* for BPhen exhibit slight shifts at the glass transition temperature *T_g_* (Figure 5b), indicating changes in the electronic structure and molecular packing. The first shoulder of *k* at approximately 4 eV disappears, while the peak at 4.5 eV shifts to 4.4 eV. Additionally, the minimum at around 5 eV moves to 4.8 eV, and a new shoulder emerges at 5.1 eV. Meanwhile, the last peak at 5.6 eV and the minimum at 5.8 eV remain nearly unchanged, with only a slight increase in amplitude. These spectral modifications suggest that the phase transition leads to molecular rearrangement and densification of the film, altering the material’s optical response. The disappearance of the 4 eV shoulder suggests that low-energy electronic transitions are suppressed, possibly due to reduced disorder and a more compact molecular arrangement. The shift of the 4.5 eV peak to 4.4 eV and the minimum at 5 eV moving to 4.8 eV indicate a reorganization of the electronic density of states, which may be caused by molecular realignment or enhanced π-π stacking interactions. The emergence of a new shoulder at 5.1 eV suggests that a previously weak or forbidden transition becomes more pronounced, likely due to changes in molecular orbital interactions. The last peak and minimum at 5.6 eV and 5.8 eV remaining nearly unchanged, with only a slight increase in amplitude, indicates that higher-energy transitions are less affected by the phase transition, as they are likely governed by deeper electronic states that are less sensitive to molecular rearrangement.

The bandgap energy *E_g_* of TAPC shifts towards lower photon energy values during its first-order phase transition (Figure 5b), indicating the reduction in electronic conjugation efficiency, likely due to weaker intermolecular interactions or increased free volume in the film. The peak at 4 eV shifts to 4.2 eV with a reduction in amplitude, the peak at 5.2 eV transforms into a minimum at 5.1 eV, and the peak at 6 eV shifts to 5.6 eV, also with decreased intensity. These spectral modifications suggest that the phase transition induces structural rearrangements and increased molecular disorder, which alter the material’s optical response. The peak amplitude reduction suggests a loss of long-range order and a decrease in oscillator strength, weakening the efficiency of electronic transitions. The transformation of the 5.2 eV peak into a minimum at 5.1 eV implies a redistribution of electronic density of states, possibly due to the reorganization of molecular orbitals. These findings highlight the impact of thermally induced phase transitions on the optical properties of TAPC thin films.

**Figure 6:**
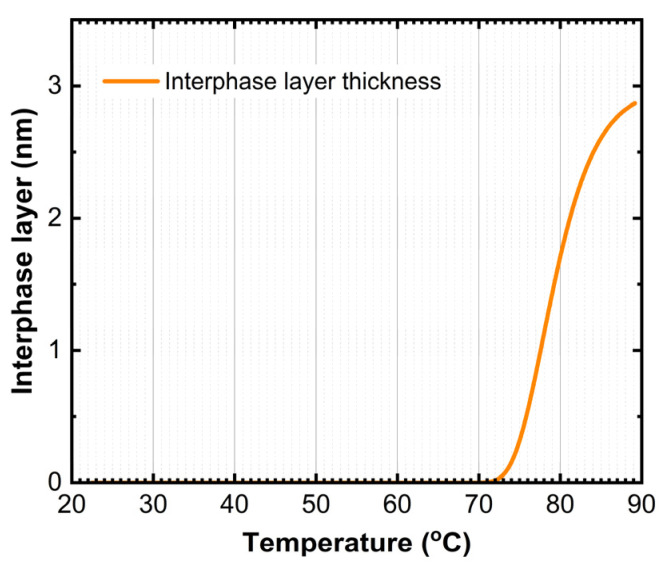
Interphase layer between BPhen and CBP thin films as a function of temperature.

The anomaly at 76 °C is likely associated with the formation of an interphase layer between BPhen and CBP. The best-fit SE model indicates that, between 75 °C and 85 °C, the thickness of this interphase region increases from 0 nm to 3 nm, suggesting the onset of interdiffusion or structural reorganization at the BPhen/CBP interface (Figure 6).

**Figure 7:**
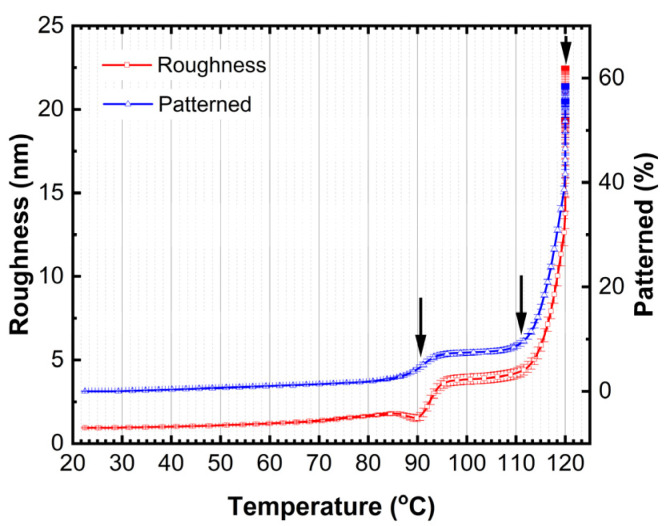
The obtained results from models (1) and (2): (1) surface roughness and (2) amount of the missing film over the ITO substrate as a function of the temperature.

**Figure 8:**
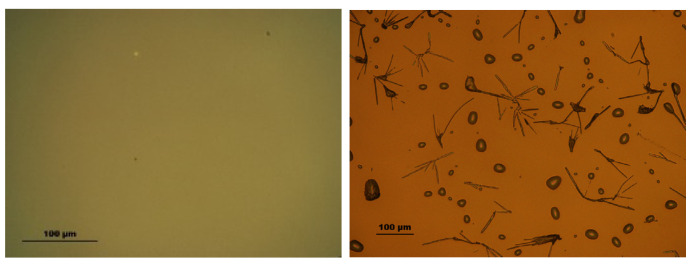
Optical microscope image of the glass/SiO_2_ buffer/ITO/TAPC/CBP/BPhen top surface before (**left**) and after (**right**) heating.

Furthermore, two models were tested: (1) surface roughness as a fitting parameter and (2) film pattering (amount of missing film over a substrate) as a fitting parameter keeping the (*n*, *k*) values of TAPC and BPhen constant. The transition seen at ~91 °C (Figure 4) is accompanied by an increase in the surface roughness (Figure 7) by applying model (1). As the temperature increases, the surface roughness continues to rise, accompanied by enhanced film patterning in the case that model (2) is used. Portions of the organic film contract into elongated islands, exposing distinct regions of the ITO substrate (Figure 8). The best-fit SE model estimates that approximately 57% of the organic film is lost from the ITO substrate, further supporting the presence of thermally induced film restructuring and material redistribution. The patterning of the thin film is attributed to reaching the CBP glass transition temperature that occurs at ~112 °C (seen as a decrease in Ψ in Figure 4), allowing increased molecular mobility and facilitating crystallization. As a result, beyond 111 °C, the material exhibits greater non-uniformity, and enhanced crystal formation throughout the structure is observed as a significant increase in patterning and roughness between the temperatures of ~112 °C and 120 °C.

The microscope image of the ITO/TAPC/CBP/BPhen thin film after heating at 120 °C reveals a highly heterogeneous surface morphology (Figure 8) as a mixture of crystalline and film-free regions. This patterning starts at around 91 °C and increases rapidly from ~112 °C as revealed from SE data (Figure 7) as a result of localized strain and morphological disruptions, contributing to the formation of sharp, elongated crystalline domains interspersed with film-free areas. The optical microscope analysis reveals that, after heating, the film coverage is (9.0 ± 0.4)%, while the exposed ITO surface accounts for (91.0 ± 0.4)% (Figure 8). These findings are in relatively good agreement with the SE results. SE yields higher film coverage values because patterning was the only fitting parameter used during the dynamic fit across the entire temperature range in model (2). The model should be improved by considering dynamic variation in the (*n*, *k*) values of all three organic thin films. Nevertheless, this simple SE model (2) confirms the observed film restructuring and partial delamination (Figure 8). The observed increase in surface roughness is very likely correlated with the rise in patterned values, as both indicate different manifestations of the same phenomenon—film shrinkage and incomplete substrate coverage. These results suggest that, as the organic layers undergo thermally induced restructuring, portions of the film contract into distinct islands, leaving exposed regions on the substrate, which can be described well by the two simple models (1) and (2).

**Figure 9:**
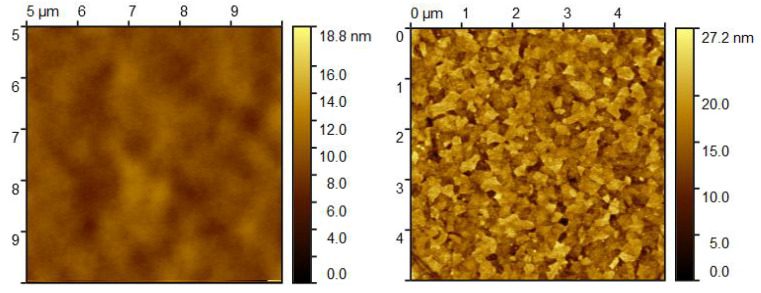
AFM image of the glass/SiO_2_ buffer/ITO/TAPC/CBP/BPhen top surface before (**left**) and after (**right**) heating.

The AFM images presented in Figure 9 show the surface morphology of the organic thin film before and after heating. Prior to heating, the root mean square (RMS) roughness *S_q_* is approximately 0.9 nm, which is in good agreement with the surface roughness values obtained from SE (Table 2). After heating, *S_q_* increases to around 3.3 nm, indicating significant surface restructuring. The AFM image prior to heating reveals a smooth, low-contrast surface with broad, flat grains—typical of amorphous organic films—reflecting the uniform, ‘liquid-like’ morphology commonly observed in as-deposited OLED layers (Figure 9, left). The AFM images also reveal that heating induces a drastic change in the microstructure, with the film transitioning from large, flat grains to a morphology dominated by smaller grains (Figure 9, right). The *S_q_* values obtained from AFM after heating do not match exactly with those determined by SE in model (1), primarily because the AFM measurements were performed in an area without empty spaces between the crystalline film and the substrate, whereas SE provides an average roughness over the entire measured surface. Nevertheless, the observed increase in roughness and crystalline grain formation is consistent with the findings from optical microscopy and SE, reinforcing the conclusion that significant morphological changes occur in the film upon heating, and that even such simple SE models like (1) and (2) can provide an insight into the physical phenomena taking place in the films during temperature increases.

**Figure 10:**
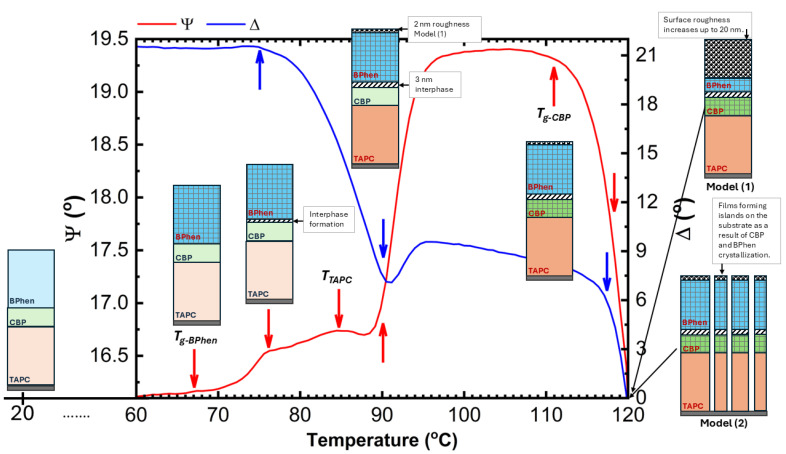
Schematic illustration of the microstructural transformations occurring in the TAPC/CBP/BPhen trilayer structure deposited on an ITO substrate as a function of temperature. The schematic is overlaid with a graph showing the main ellipsometric angles (Ψ and Δ) versus temperature, highlighting how the microstructural changes are reflected in the temperature-dependent behavior of Ψ and Δ.

Figure 10 presents a schematic illustration of the microstructural transformations occurring in TAPC/CBP/BPhen on an ITO substrate, as a function of the temperature. The optical response of (Ψ; Δ) is plotted as well to provide a better understanding of the phase transition temperatures. At room temperature (~22 °C), the TAPC, CBP, and BPhen layers exist in an isotropic state, maintaining their room-temperature optical properties. As the temperature increases, the glass transition of BPhen occurs at ~66.5 °C, leading to molecular rearrangement and the formation of a more ordered phase, which is represented as a checkered dark blue structure in the diagram. Upon further heating, at ~76 °C, an interphase layer starts forming between CBP and BPhen, indicating molecular interdiffusion or partial reorganization at the interface. The first-order phase transition of TAPC at ~84.5 °C is depicted as a dark orange layer, marking a structural transformation in TAPC, which is accompanied by changes in its optical dispersion properties. Additionally, at around ~91 °C, the increase in the surface roughness is associated with BPhen crystallization. At ~112 °C, CBP undergoes its glass transition, represented by a checkered dark green structure, leading to increased molecular mobility and potential densification or reorganization. Finally, at ~112–120 °C, a drastic morphological change occurs, with the formation of islands on the substrate as a result of CBP and BPhen crystallization. This phase separation and restructuring can be described by (1) a significant increase in roughness (~20 nm) or (2) a reduction in continuous film coverage, as reflected in the sharp changes in the Ψ and Δ spectra (Figure 3 and Figure 4). Certain fitting parameters describing the three-layer structure near *T_a_* are summarized in Table 2. This comprehensive thermal evolution of the TAPC/CBP/BPhen system highlights the importance of thermal stability and phase behavior in organic multilayer structures, with implications for OLED performance, degradation mechanisms, and device reliability. These results also demonstrate that direct SE measurements can identify critical temperatures in TAPC/CBP/BPhen OLED structures and that significant morphological changes can be described using the simple models (1) and (2).

## 4. Conclusions

This study provides a comprehensive analysis of thermally induced phase transitions in multilayer OLED thin films, utilizing in situ SE to monitor structural and optical changes under controlled heating. Key anomalies were detected at 66.5 °C, 76.3 °C, 84.5 °C, 91 °C, and 112 °C directly in (Ψ, Δ) spectra, corresponding to significant morphological and optical transformations in BPhen, CBP, and TAPC layers.

The findings confirm that the glass transition of BPhen (66.5 °C) and CBP (111 °C), along with the first-order phase transition of TAPC (84.5 °C), affect the material’s refractive index, extinction coefficient, and surface morphology. The (*n*; *k*) dispersion curves for TAPC and BPhen before and after their respective phase transition temperatures are also presented in this work. Additionally, the formation of interphase layer between BPhen and CBP was detected around 76 °C, while at 91 °C, increased roughness and restructuring of BPhen were observed. The glass transition temperature of CBP manifests as the decrease in Ψ at the temperature of 112 °C. Due to low thickness of CBP, it was not possible to obtain (*n*, *k*) at *T_g_*. At 112 °C, a substantial film shrinkage and patterning resulted in the formation of elongated crystalline domains, reducing the film coverage down to approximately 57%, as obtained by the simple SE model, and down to 91% confirmed by optical microscopy.

This study also presents a schematic illustration of the morphological and structural changes occurring in the TAPC/CBP/BPhen multilayer structure during thermo-optical evolution. These illustrations provide a visual representation of the key phase transitions, interphase formation, and crystallization effects observed in the experiment.

These results highlight the critical role of phase transitions and interfacial interactions in OLED stability and degradation kinetics. The correlation between SE measurements, AFM, and optical microscopy shows the importance of SE on in situ studies of OLEDs. By understanding the interplay between phase transitions, interphase formation, and morphological evolution, this study contributes to the development of more stable and durable OLED devices.

## Figures and Tables

**Table 1:** Temperature anomalies *T_a_* observed in ellipsometric (Ψ; Δ) spectra: comparison with the literature, sensitive parameters where *T_a_* was observed, and the description of phase transition. The ↓↑ indicates if the sensitive parameters increase or decrease at *T_a_*.

*T_a_* (°C)		Parameter	Material	Description of Phase Transition
This Work	Ref.			
66.5 ± 0.5	66 [18]	Ψ↑	BPhen	*T_g_* of BPhen
76.3 ± 0.5	-	Ψ↑, Δ↓	-	3 nm interphase layer creation between BPhen and CBP layers
84.5 ± 0.5	84.5 [19]	Ψ↓	TAPC	The 1st-order reversible phase transition of TAPC
91 ± 1	-	Ψ↑, Δ↓, roughness ↑	-	Increase in the surface roughness due to BPhen crystallization
112 ± 2	111.85 [20]	Ψ↓, Δ↓, roughness ↑, patterned ↑	CBP	*T_g_* of CBP Shrinking of the multilayer film structure die

**Table 2:** Certain fitting parameters at specific temperatures near *T_a_*: surface roughness *S_r_*; BPhen; interphase; CBP, TAPC, ITO, and SiO_2_ buffer layer thicknesses *d*, band gaps *E_g_*, and film patterning (amount of the film covering the ITO substrate).

	T_a_	22 °C	67 °C	77 °C	85 °C	91 °C	112 °C	120 °C
Parameters								
**S_r_ (nm)**		0.93 ± 0.06	1.3 ± 0.05	1.6 ± 0.06	1.8 ± 0.1	1.8 ± 0.2	(4.3–4.8) ± 0.3	21.0 ± 1.5
**d_BPhen_ (nm)**		32.54 ± 0.06						
**d_interf_ (nm)**		-		0.8 ± 0.02	2.5 ± 0.02			
**d_CBP_ (nm)**		9.44 ± 0.05						
**d_TAPC_ (nm)**		44.95 ± 0.07						
**E_gBPhen_ (eV)**		3.86 ± 0.02	3.84 ± 0.07					
**E_gCBP_ (eV)**		2.89 ± 0.02						
**E_gTAPC_ (eV)**		3.66 ± 0.02			3.49 ± 0.07			
**Patterning (%)**		-	1.3 ± 0.1	1.6 ± 0.1	2.5 ± 0.2	5.1 ± 0.4	(9.6–11.4) ± 0.5	57.5 ± 1.4
**d_ITO_ (nm)**		102.56 ± 0.03						
**d_SiO2_ (nm)**		34.50 ± 0.52						

## Data Availability

The data presented in this study are openly available in [Zenodo] at [https://zenodo.org/records/15055400] providing (*n*, *k*) of ITO, SiO_2_ buffer and soda lime glass, https://zenodo.org/records/15051868] providing (*n*, *k*) data sets of OLEDs at room temperature, and at [https://zenodo.org/records/15211713] providing (*n*, *k*) for TAPC and BPhen just after phase transition temperatures (at 85 °C for TAPC and at 67 °C for BPhen). The authors of this would like to republish selected Figure 1, Figure 2, Figure 3 and Figure 4 in the proceedings of the OPAL 2025 conference, to be held in May 2025, in combination with additional and complementary results recently obtained on these samples.

## Abbreviations

The following abbreviations are used in this manuscript:

BPhen	Bathophenanthroline
CBP	(4,4′-Bis(N-carbazolyl)-1,1′-biphenyl)
EML	Emissive layer
ETL	Electron transport layer
GO	Gaussian oscillator
HJPS	Herzinger–Johs Parameterized Semiconductor
ITO	Indium tin oxide
NIR	Near-infrared region
OLED	Organic light-emitting diode
SE	Spectroscopic ellipsometry
TAPC	(4,4′-Cyclohexylidenebis [N,N]-bis(4-methylphenyl)benzenamine
UV	Ultraviolet spectral region
VIS	Visible spectral region
